# Pilot and feasibility studies in exercise, physical activity, or rehabilitation research

**DOI:** 10.1186/s40814-018-0326-0

**Published:** 2018-08-14

**Authors:** Rasha El-Kotob, Lora M. Giangregorio

**Affiliations:** 1 Department of Kinesiology, University of Waterloo, 200 University Avenue West, Waterloo, ON N2L 3G1 Canada; 2 Brain and Spinal Cord Rehabilitation Program, Toronto Rehabilitation Institute, University Health Network, 520 Sutherland Dr, Toronto, ON M4G 3V9 Canada; 3 Schlegel-University of Waterloo Research Institute for Aging, University of Waterloo, 250 Laurelwood Dr, Waterloo, ON N2J 0E2 Canada

**Keywords:** Pilot, Feasibility, Physical activity, Exercise

## Abstract

**Background:**

Clinical trials of physical activity and rehabilitation interventions can be challenging. Pilot or feasibility studies can be conducted prior to a definitive randomized controlled trial (RCT), to improve the chances of conducting a high-quality RCT of a physical activity intervention.

**Main body:**

Physical activity interventions or trials present unique challenges at the population, intervention, comparator and outcome levels. At each level, we present guidance for researchers on the design considerations for pilot or feasibility studies of physical activity interventions. When it comes to defining study population, physical activity trials often exclude participants with certain health conditions or other characteristics (e.g., age, gender) because of uncertainty of the safety of the exercise intervention or presumed differences in responsiveness, at the expense of trial generalizability. A pilot trial could help investigators determine refined inclusion and exclusion criteria to balance safety, adequate recruitment, and generalizability. At the intervention level, because exercise can be a complex intervention, pilot trials allow investigators to evaluate participant adherence and instructor fidelity to the intervention and participant experience. At the comparator level, control group dissatisfaction and post-randomization drop-out can occur, because of the desire to be randomized to the exercise group, and the difficulty with blinding to group allocation; an active control or deception could be used. Finally, at the outcome level, there should be an emphasis on the pilot or feasibility outcomes such as recruitment rate, adherence to exercise, and retention or fidelity, than the efficacy of the exercise intervention.

**Conclusion:**

Physical activity and rehabilitation researchers can use pilot and feasibility studies to enhance the rigor of future trials, while also publishing the results of their pilot work to move the field forward. Researchers in this field are encouraged to use published reporting guidelines for pilot and feasibility studies and to consider the challenges discussed in this paper.

## Background

Randomized controlled trials (RCTs) of exercise, rehabilitation or physical activity interventions (from here, collectively called physical activity interventions) are challenging and resource-intensive. Physical activity interventions often require supervision by exercise or rehabilitation professionals [1]. Participants have to dedicate time and effort to participate and achieve a prescribed frequency, intensity, type, and duration of physical activity; adherence is a challenge and blinding to group allocation is often impossible [1, 2]. Outcomes include performance-based measures such as gait speed, aerobic capacity, muscular strength tests, or functional tests of balance and mobility, such as the Timed Up and Go or timed chair stands, thus requiring in-person follow-up and good retention [1]. There is increasing emphasis on reducing bias and improving the quality of clinical trials in the physical activity realm, as demonstrated by the publication of reporting guidelines specific to such interventions [1, 2]. One way to do this is to work out the uncertainties of the trial design beforehand. A feasibility study can be used to determine whether a future main study can be done, while a pilot study is a subset of a feasibility study that resembles the main trial but with the overall aim to assess the feasibility rather than the effectiveness of a study protocol [3]. Therefore pilot or feasibility studies can be conducted prior to a definitive RCT, to improve the chances of success in conducting a high-quality RCT of a physical activity intervention.

Reasons for conducting pilot or feasibility studies can fall under four categories: to inform process (e.g., feasibility of recruitment, retention, intervention adherence), to understand resource requirements (e.g., time and budget issues), to inform management (e.g., personnel challenges, data collection or organization), and to advance scientific inquiry (e.g., intervention safety, appropriate dose, potential treatment effect) [4]. Inferences about efficacy cannot be made from a physical activity study that is underpowered due to unsuccessful recruitment and retention, and it will be difficult to observe an effect if there is poor adherence. Therefore, instead of investing in underpowered hypothesis-testing studies, conducting exploratory pilot or feasibility studies with clear a priori criteria for success can better inform future efforts. If a pilot study turned out not to be feasible it is not considered a failed pilot study but rather a success, since the team has avoided spending resources on a large study that will not succeed [4].

Many pilot and feasibility studies examining physical interventions do not have clear a priori criteria for success and often focus on efficacy outcomes of the intervention rather than feasibility [5–9]. Researchers with training in exercise, physical activity, or rehabilitation research may not always have received formal training in the design and conduct of clinical trials nor have expertise in how to design and interpret a pilot study. We discuss pilot and feasibility studies in the context of physical activity interventions with the overall aim of promoting the successful use of pilot and feasibility studies in physical activity research. Challenges unique to physical activity pilot/feasibility studies will be presented under the categories population, intervention, comparator, and outcome (PICO). Example challenges are summarized in Table 1, and the examples used are summarized in Table 2.

**Table 1:** Considerations for pilot and feasibility studies of physical activity interventions

Population	Intervention	Comparator	Outcome
• Balance safety, ability to complete intervention or assess outcomes, and generalizability when selecting inclusion/exclusion criteria • Willingness to be randomized to the non-exercise group • How to assess baseline physical activity level and define inclusion/exclusion criteria, e.g., at what frequency, intensity, time, and type to exclude because they are too active	• Participant and instructor fidelity • What type of personnel is required for safe intervention delivery and participant assessment? • Ability and willingness of the participants to understand and adhere to the exercise program • How to measure adherence, especially for unsupervised exercise • Adherence tends to decrease over time—what strategies maximize adherence to intervention? • Exercise setting is accessible, does not create barriers that influence feasibility, and potential generalizability	• Difficult to create a placebo or to blind participants to group allocation • Usual care or attention control group provides equal attention, but because of lack of blinding, may still create challenges with recruitment, retention or potential for bias • Post-randomization drop-out rates may be unequal if control group is dissatisfied	• Emphasis on feasibility objectives and not secondary outcome measures • Must have a priori criteria for success • How to impute missing data if data not missing at random, e.g., drop-out because randomized to control, individuals with impaired mobility may not be able to complete performance based measures at baseline

**Table 2:** Examples of pilot and feasibility studies involving physical activity

Authors	Population	Intervention	Comparison	Outcome	Time	Criteria for Success
Connolly et al. [7]	In-patients with intensive care unit-acquired weakness	Exercise-based rehabilitation program (EBRP)	Standard care	Recruitment, adherence to the EBRP, adverse events, patient exercise time per session, educational sessions, patient acceptability, exercise capacity, and health-related quality of life.	12 weeks	Unspecified
Granger et al. [15]	In-patients undergoing lung resection for cancer	Standard care physiotherapy, plus twice daily in-patient exercise program, plus twice weekly out-patient exercise program, plus unsupervised home based exercise program* *Randomization occurred on the first day after surgery	Standard care physiotherapy* *Randomization occurred on the first day after surgery	Safety: Number of adverse events during exercise testing and exercise training. Feasibility: Recruitment rate, consent rate, number of inpatient exercise sessions delivered, and participant attendance at outpatient sessions. Secondary outcomes included functional capacity, functional mobility, and health-related quality of life.	10 weeks	Unspecified
Patten et al. [10]	Females, 18–55 years of age, smoked at least 10 cigarettes per day for at least the past year, willing to quit, currently depressed as defined by the Centre for Epidemiological Studies Depression Scale (cutoff score of at least 16), and not meeting the American College of Sports Medicine exercise guidelines.	Evidence-based cessation counseling plus exercise intervention	Evidence-based cessation counseling (exercise was not discussed) plus health education	Feasibility: participant recruitment, study retention, and treatment adherence. In addition, measured smoking status, cardiorespiratory fitness, physical activity, body mass index, physical activity, and depressive symptoms.	12 weeks	Unspecified
Barker et al. [8]	Community-dwelling older adults (≥ 60 years of age) at risk of sustaining a fall injury and able to climb 10 stairs independently.	A 60-min Pilates class twice a week for 12 weeks delivered in a group setting plus a 20-min tailored home-based exercises to complete on a daily basis.	20-min tailored home-based exercises to complete on a daily basis.	Feasibility: acceptability was measured based on recruitment, retention, intervention adherence, and participant experience survey. Safety: adverse events. Potential effectiveness: fall, fall injury and injurious fall rates, standing balance, lower limb strength, and flexibility.	24 weeks	Unspecified
Suttanon et al. [11]	Community-dwelling older adults diagnosed with mild to moderate Alzheimer’s disease who could walk outdoors with minimal support (no more than a single-point stick). Also, they cannot have any serious orthopedic conditions or major neurological disorders that could limit their functional mobility.	Individualized home-based exercise program with intermittent supervision	Home-based education program	Feasibility: adverse events, balance, mobility, falls, and falls risk	24 weeks	Unspecified
Giangregorio et al. [9]	Women who are 65 years of age or older with a vertebral fracture.	Home-based exercise and behavioral counseling with intermittent supervision	Same number and duration of visits and calls but were not exercise related.	Feasibility: recruitment, retention and exercise adherence. Secondary outcomes: fractures, falls, posture, physical performance, quality of life, pain, health service use, behavior change variables and fall self-efficacy.	52 weeks	Recruitment: recruit 20 participants per site. Retention: ≥ 75% of the study sample completes visit 2. Adherence: ≥ 60% of the number of exercises to be completed at the 1-year follow-up.

## Challenges to physical activity research

### Population

Pilot or feasibility studies can be used to determine how many participants are potentially eligible and how many are excluded and why, while also considering current physical activity levels and providing realistic screening to recruitment ratios in the population of interest, so that when planning a larger trial, researchers have a good estimate of how long it will take or if they need to recruit more sites or broaden their inclusion criteria.

#### Inclusion and exclusion criteria

Considerations specific to physical activity interventions include determining the inclusion/exclusion criteria related to safe participation at the required frequency, intensity, duration, and type of physical activity and how to exclude those who are already participating in a comparable physical activity intervention. A basic criterion is to exclude individuals who meet any of the American College of Sports Medicine (ACSM) relative or absolute contraindications to exercise [10]. Participant screening should include current physical activity status; signs and symptoms of cardiovascular, metabolic, or renal disease; and capacity to complete prescribed physical activity given the level of supervision [10]. For example, the risk of exercise-related cardiovascular events is greater among inactive participants who engage in physical activity at vigorous intensities [10–12]. Screening could also include tools like the “Get Active Questionnaire” [13]. Researchers often err on the side of caution and create eligibility criteria for physical activity trials that are too restrictive, or not generalizable to real world practice. For instance, an exercise-based rehabilitation intervention study comprised of participants with intensive care unit-acquired weakness had strict eligibility criteria, and this resulted in a low ratio of included to excluded participants [5]. A pilot or feasibility study could have been used as a first step to refine the inclusion and exclusion criteria for the physical activity intervention. If the proposed definitive trial is a multicenter trial, one should evaluate feasibility of recruitment at multiple centers as opposed to assessing feasibility at a single site.

#### Baseline physical activity level

Physical activity trials often aim to recruit individuals who are physically inactive, but individuals who express interest may already participate in some activity. A pilot or feasibility trial could evaluate the characteristics of those who express interest, to determine potential selection bias, including baseline physical activity level. One approach is to consider what the “active ingredients” of the intervention are and exclude those already participating in activities that contain those active ingredients. For example, if one is examining the efficacy of a moderate-high resistance training intervention on muscle strength, should a person who does yoga and walks every day be eligible? For multicomponent interventions, the screening becomes more complex. Our recent trial of home exercise in women with vertebral fractures included balance training, posture training and resistance exercises, and daily aerobic physical activity; we excluded individuals with exercise participation ≥ 3 times per week that addressed ≥ 2 of the domains [14]. Based on the inclusion criteria, individuals who walked daily were eligible because they were only participating in one of the domains (i.e., daily aerobic physical activity), and a key focus of the intervention was resistance and balance training. Researchers should use a physical activity screening tool validated for the population of interest, e.g., International Physical Activity Questionnaire, the Rapid Assessment of Physical Activity, or the Global Physical Activity Questionnaire.

### Intervention

A unique challenge in physical activity trials that can be evaluated in pilot/feasibility studies is feasibility of intervention implementation including fidelity of intervention delivery, assessment of intervention adherence, and consideration of factors that might influence adherence.

#### Fidelity of intervention delivery

There are two types of treatment fidelity that can be evaluated in pilot/feasibility studies: participant fidelity and instructor fidelity. Participant fidelity refers to whether the participant completes the prescribed activities or exercises as required by the protocol (e.g., the right frequency, intensity, duration, and type), and instructor fidelity refers to whether the activities or exercises are prescribed, tailored, or demonstrated in accordance with the protocol. In a study by Patten et al. ACSM-certified trainers delivered the exercise interventions after 6 h of training on the study protocol [7]; when 15% of the sessions were randomly audited, coach adherence to the training manual was 95%, representing high instructor fidelity [7]. Suttanon et al. involved the caregivers in a home-based exercise program designed for older adults with Alzheimer’s disease [9]. Some caregivers indicated that they had competing demands or had health conditions that prevented them from continuously supporting the exercise program. A pilot trial could record modifications made (or that need to be made) to the intervention to inform a future trial.

Another consideration is that physical activity interventions require that participants not only understand the study but also to complete assessments or to adhere to the exercise intervention, particularly when participants are required to understand and execute exercises with proper form while unsupervised [5, 8]. A pilot study might consider testing various methods for evaluating informed consent or willingness to participate in study activities. A simple way to evaluate whether the participants fully understand or can complete tasks or exercises might include describing the study and asking the participant to repeat what they are being asked to do in their own words. If the focus of the trial is explanatory, it is desirable to only enroll participants who are likely to adhere to the intervention, one could consider a “faintness of heart” period [15, 16]. For example, potential participants could be asked to complete one exercise or task daily and record it in an exercise adherence log for 2 weeks. Those who fail to return the log would lose eligibility, and those who return a completed log could then be randomized. Or, adherence could be a key outcome of a pilot trial.

#### Assessment of intervention adherence

A pilot or feasibility trial is ideal for assessing whether participants will adhere to an intervention with predefined criteria for success that are aligned with a hypothesized minimum dose. The criteria for success could be defined based on a dose response study, or prior work that informs what is realistic or what might be a minimum effective dose. If adherence is a primary feasibility outcome, the methods for assessing adherence must be rigorous, a goal that is easy to achieve in supervised interventions, but less so in unsupervised ones. To monitor exercise adherence for the latter, participants are often asked to complete a self-reported exercise diary, which is subject to social desirability bias and non-response bias. For example, Suttanon et al. indicated that only two thirds of monthly exercise logs were completed by the end of the study [9]. Completing calendars are considered burdensome by participants and could lead to study withdrawal [8]. Activity monitors can also be used to assess physical activity levels but have limited utility in interventions that do not involve walking or running; they do not capture resistance training, cycling, balance training, or swimming, for example. A pilot/feasibility trial could also consider the feasibility of compliance with physical activity monitors. For example, Patten et al. found that the majority of the participants did not wear the device provided [7]. Since participants and those delivering interventions may not be blind to group allocation, blinding assessors becomes more important in physical activity trials to reduce detection bias. Physical activity trials often include physical activity outcomes or adherence, or other outcomes (e.g., exercise self-efficacy) that when assessed, could reveal group allocation. Therefore, it would be wise to have separate assessors not blind to group allocation evaluate these outcomes so as to ensure that assessors examining non-exercise related outcomes remain blind to group allocation.

#### Factors influencing intervention adherence

A pilot or feasibility study should also explore factors that influence adherence, including intervention type and setting, participant satisfaction or experience measures, or conduct exit interviews to gather input on their overall experience with the study, or the intervention. The method of delivery, group versus individual exercise program, as well as the setting, such as community setting or at home, can influence adherence to the physical activity intervention [9]. Home-based exercise interventions do not involve the planning of transportation or scheduling difficulties, but unsupervised or intermittently supervised interventions may compromise adherence or intervention fidelity. Intermittent supervision by means of home visits or telephone calls may help but add cost (e.g., time, travel), complexity, and variability in terms of intervention fidelity. The number of home visits or telephone calls should be determined when designing the exercise intervention, and the proximity of persons delivering the intervention to the participants should be considered. A cross-sectional study examined exercise adherence to strength training in older women and showed that those who were older, had greater physical activity level, better perceived overall health, and were supervised by a well-trained professional were more likely to adhere to their exercise regimen [17]. To enhance adherence, some studies include exercise-related behavior interventions that involve goal setting, time management, and overcoming barriers [18, 19]. Behavior intervention strategies, however, do not always improve exercise retention or adherence [20]. Nevertheless, to ensure effective counseling, the strategies employed should be standardized and completed by trained personnel [21]. When it comes to physical activity interventions, there is substantial scope for assessing feasibility in advance of a large trial, including intervention fidelity, participant experience and adherence.

### Comparator

An ideal comparison group is a placebo, such that the participants are blinded to whether they are in the active treatment group. However, a placebo control is difficult or near impossible for physical activity interventions, resulting in the potential for performance bias (i.e., that knowledge of the intervention received will influence outcomes). Further, what often attracts potential participants is the opportunity to be in the exercise group. A feasibility study should assess contamination or post-randomization drop-out rates in the comparison group relative to the intervention group. As an example, Barker et al. experienced a 23% drop-out rate in the control group, which was higher than anticipated, versus a 14% drop-out rate in the exercise group [8]. Participants lost interest after being allocated to the control group, and it was the most commonly reported reason for study withdrawal [8]. Individuals randomized to control who really want to start exercise may seek an exercise program outside of the study; a pilot study could monitor whether this is an issue. Compensatory strategies might include an attention control or an active control that is attractive to participants, with or without deception (e.g., tell participants the study is comparing two exercise interventions). For instance, Giangregorio et al. and Suttanon et al. made sure that the control group received the same number and duration of home visits and phone calls [9, 14]. Patten et al. made sure that all participants received identical evidence-based smoking cessation counseling and added exercise counseling in the intervention group [7]. Studies of challenging balance interventions could provide seated or lying exercises as the comparator [8]. Therefore, an alternative exercise intervention can be selected for the comparator group as long as it does not have an effect on the outcome of interest. A feasibility study could assess participant adherence to or acceptance of the attention/active control, and whether it prevented contamination or post-randomization drop out.

### Outcome

Feasibility outcomes could include recruitment rate [5–8, 14], consent rate [6], adherence to the physical activity intervention [5–8, 14], study retention [7, 8, 14], adverse events [5, 6, 8, 9], and participant experience or satisfaction [5, 7, 8, 14]. The decision to move from a pilot/feasibility study to a full trial should be based on the feasibility objectives and not the secondary outcome measures. Sometimes, emphasis is placed on efficacy outcomes for which the pilot/feasibility trial was not designed or powered to assess. Outcomes related to the efficacy of the intervention can be measured, but should be treated as exploratory, and not hypothesis-testing. However, one could explore the feasibility of measuring the outcomes that will be used to assess efficacy in a future trial, e.g., potential for ceiling or floor effects, the time taken to complete assessments, and extent to which data is missing. Intention to treat analyses require imputation of missing data, but sometimes data are not missing at random e.g., because of difficulties completing performance-based measures, or higher drop-out rate in controls because of dissatisfaction with group allocation. All pilot or feasibility studies should have a priori criteria for success related to the primary feasibility questions, to inform unbiased conclusions as to whether it is feasible for the pilot study to become a main study [4]. Unfortunately, however, many pilot and feasibility studies of physical activity interventions fail to specify criteria for success (Table 2), and instead make vague claims about high completion rates, good attendance, an absence of adverse events, or strong patient acceptability. In summary, pilot or feasibility outcomes should be measuring whether a future trial is realistic or feasible. Pilot or feasibility trials should not be used to provide a conclusive estimate as to whether an intervention is effective.

#### Reporting of pilot and feasibility trials of physical activity interventions

High-quality pilot and feasibility trials have the potential to advance the future success of clinical trials in the physical activity realm. They form the foundation for exceptional grant proposals to secure funds for large trials, as they can increase reviewer confidence regarding the team’s capacity to do the work. Further, they can still contribute to academic productivity if they are published. Although pilot or feasibility studies are informative, they are not often published due to emphasis on efficacy outcomes or they lack clear feasibility objectives and do not specify criteria for success (Table 2) [4]. In addition, there are misconceptions regarding what is considered a pilot study. For example, studies performed with limited funding, in a short time frame, or with restricted resources, are not reasons a study should be called a pilot [4]. Given the complex nature of physical activity interventions, pilot and feasibility studies in this field should be more abundant. Grant review panels and journals, however, will not recognize the value of pilot studies without rigor in design, conduct, and reporting. Accordingly, the Consolidated Standards of Reporting Trials (CONSORT) group has now created extensions specific to the reporting of pilot and feasibility trials [2] and of non-pharmacological interventions [22]. Adhering to the CONSORT 26-item checklist can improve reporting and can also help in designing and evaluating future trials [2]. Journals are increasingly becoming receptive to high quality pilot trials, and the journal Pilot and Feasibility Studies is an ideal destination.

## Conclusions

Pilot and feasibility studies of physical activity interventions can inform the design and conduct of large, definitive trials by testing how the trial processes work together and by evaluating feasibility of recruitment, retention, adherence, intervention delivery, outcome assessment, or any other aspect of the trial that could compromise its success. Feasibility issues unique to physical activity trials include the challenge of blinding of group allocation, retaining the comparison group, participant and instructor fidelity, and being able to recruit people not already active enough, but interested enough in becoming active to sign up for the study. A pilot trial should have clear objectives and a priori criteria for success and should be designed, conducted, and reported using the same standards as any high-quality randomized controlled trial. Overall, pilot and feasibility studies can ensure that scarce research money and researcher efforts are being invested in large trials that can provide definitive answers to important research questions.

## Abbreviations

ACSM: American College of Sports Medicine
CONSORT: Consolidated standards of reporting trials
PICO: Population, intervention, comparator, and outcome
RCT: Randomized controlled trials
